# Inherited Thoracic Aortic Disease: New Insights and Translational Targets

**DOI:** 10.1161/CIRCULATIONAHA.119.043756

**Published:** 2020-05-11

**Authors:** Alexander J. Fletcher, Maaz B.J. Syed, Timothy J. Aitman, David E. Newby, Niki L. Walker

**Affiliations:** 1 University of Edinburgh Centre for Cardiovascular Science, Royal Infirmary of Edinburgh, United Kingdom (A.J.F., M.B.J.S., D.E.N., N.L.W.).; 2 Centre for Genomics and Experimental Medicine, MRC Institute of Genetics and Molecular Medicine, University of Edinburgh, United Kingdom (T.J.A.).; 3 Scottish Adult Congenital Heart Disease Service, Golden Jubilee National Hospital, Clydebank, Glasgow, United Kingdom (N.L.W.).

**Keywords:** aneurysm, dissecting, aortic aneurysm, bicuspid aortic valve, biomarkers, Marfan syndrome

## Abstract

Inherited thoracic aortopathies denote a group of congenital conditions that predispose to disease of the thoracic aorta. Aortic wall weakness and abnormal aortic hemodynamic profiles predispose these patients to dilatation of the thoracic aorta, which is generally silent but can precipitate aortic dissection or rupture with devastating and often fatal consequences. Current strategies to assess the future risk of aortic dissection or rupture are based primarily on monitoring aortic diameter. However, diameter alone is a poor predictor of risk, with many patients experiencing dissection or rupture below current intervention thresholds. Developing tools that improve the risk assessment of those with aortopathy is internationally regarded as a research priority. A robust understanding of the molecular pathways that lead to aortic wall weakness is required to identify biomarkers and therapeutic targets that could improve patient management. Here, we summarize the current understanding of the genetically determined mechanisms underlying inherited aortopathies and critically appraise the available blood biomarkers, imaging techniques, and therapeutic targets that have shown promise for improving the management of patients with these important and potentially fatal conditions.

The aorta is the largest artery in the body, beginning at the aortic valve of the heart and finishing at its bifurcation point in the pelvis. The aorta acts as a conduit for blood by connecting the heart to the major arteries, ensuring blood flow to all the vital organs of the body. Being the most proximal artery to the heart, the thoracic aorta, the portion of the aorta contained within the chest, withstands the highest pressure and wall stresses through >30 million pressure cycles a year. The thoracic aortic wall must therefore function as a robust biomechanical structure, loading and unloading the cardiac volume efficiently and reliably throughout life. To achieve this, the molecular and biomechanical environment of the aortic wall is monitored and stabilized through a complex interplay between vascular wall cells and the extracellular matrix. Unfortunately, this robust homeostatic process can be disturbed through a variety of disease processes, collectively described as thoracic aortopathies, in which the thoracic aortic wall becomes weak and vulnerable. Embryologically, the descending thoracic and abdominal aorta arises from the mesoderm, whereas the aortic root and ascending aorta are derived from the neural crest, leading to differences in gene expression and behavior of vascular smooth muscle cells in each region.^1^ Furthermore, the aortic wall composition, mechanisms of aneurysm formation, and risk factors for their progression and complications are different in the 2 areas. Consequently, although there are distinct similarities, it is prudent to consider thoracic and abdominal aortic disease processes separately. Here, we focus on the disease processes of thoracic aortopathy.

## The Need to Improve Detection and Management of Inherited Thoracic Aortopathy

Inherited thoracic aortopathies denote conditions in which abnormalities lead to thoracic aortic wall weakness or abnormal aortic hemodynamic profiles, predisposing patients to aortic dilatation, aneurysm formation, and acute aortic complications. Degradation of the components of the thoracic aortic wall can also occur as part of acquired processes, for example, associated with aging or high blood pressure, factors that potentially compound genetically influenced thoracic aortic wall weakness. Patients with inherited aortopathy are at substantially increased risk of acute aortic complications, including aortic dissection or rupture, carrying with it major mortality risk. Because the outcomes from elective surgery are superior to those associated with emergency repair once dissection has occurred, current international guidelines advocate a focus on recognizing those at risk early, regular monitoring with imaging, and prophylactically replacing the diseased aortic segment once a threshold diameter is reached (Table 1).^2–8^ Even with these guidelines and robust monitoring, many patients with inherited thoracic aortopathy still experience aortic dissection or rupture while still below the threshold diameter, whereas others may undergo surgery for thoracic aortic aneurysms that are unlikely to dissect or rupture.^9,10^ There is therefore universal agreement that better identification and risk stratification of thoracic aortopathy are urgently required. Indeed, a substantial body of research has been dedicated to exploring and developing novel solutions to this problem. This review summarizes the epidemiology and underlying pathological processes associated with inherited aortopathy, outlines current management strategies, and examines the evidence supporting promising therapeutic targets and emerging biomarkers for these high-risk aortopathies.

**Table 1.**
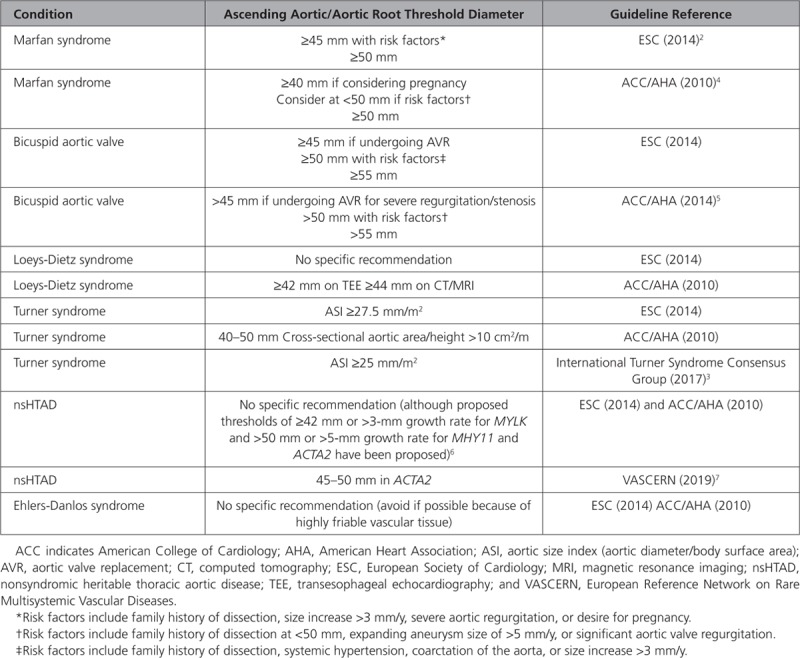
Summary of the Current Ascending Thoracic Aortic Aneurysm Diameter Thresholds for Considering Surgical Intervention

## Anatomy, Structure, and Function of the Thoracic Aorta

**Figure 1.**
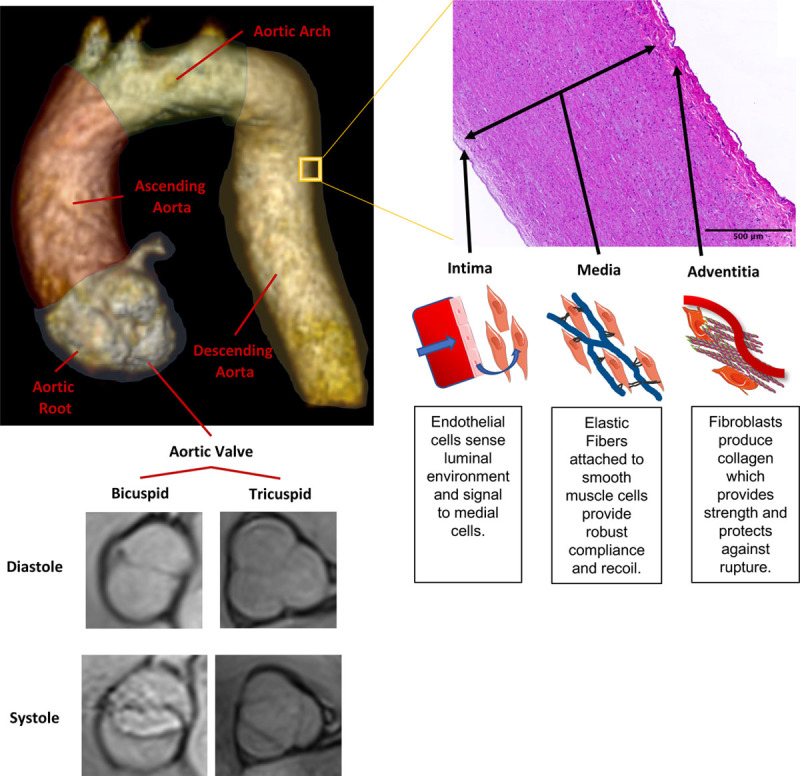
**Anatomy and histology of the thoracic aorta.** Schematic representation of the thoracic aorta three-dimensional (3D) rendering from a contrast-enhanced magnetic resonance imaging (MRI) angiogram and both and tricuspid aortic valve from taken from MRI cine imaging (images captured on 3T Biograph mMR, Siemens, Erlangen, Germany; 3D rendering performed in Horos GNU Lesser General Public License, version 3) and a histological sample of the aortic wall stained with hematoxylin and eosin. The 3 layers of the aortic wall are demonstrated, and a basic representation of the composition for each layer is demonstrated.

The thoracic aorta begins at the aortic valve, finishes as the aorta passes through the diaphragm, and is made up of 4 sections; the aortic root, which encompasses the coronary arteries that supply the heart; the tubular ascending aorta, which runs from the sinotubular junction to the origin of the brachiocephalic artery; the aortic arch, which contains the head and neck vessels and finishes just after the left subclavian artery; and the descending thoracic aorta, which ends at the level of the diaphragm (Figure 1). Inherited aortopathies can affect any part of the thoracic aorta but most commonly affect the aortic root or ascending aorta.

Aortic dilatation in adults has classically referred to increased absolute aortic diameters based on reference thresholds, typically >40 mm, although a range of thresholds have been proposed and referenced in research.^11^ However, concerns about increased risk of dissection occurring at lower gross diameters in smaller patients have led to wider use of aortic diameters adjusted for age, body surface area (aortic size index), or height (aortic height index), with aortic dilatation defined as an indexed aortic diameter *z* score >2.0 compared with reference populations.^12,13^ The terms “thoracic aortic aneurysm” and “aortopathy” are often used interchangeably with aortic dilatation, although thoracic aortic aneurysm refers to an increase in diameter of >50%, a measure not commonly used accurately, whereas aortopathy is a generic description of a pathological process affecting the aorta, which can be a useful descriptor of a diseased aorta regardless of its diameter.

The aortic wall is histologically made up of 3 sections (Figure 1). The intima is the deepest, in direct contact with the lumen, and contains a lining of endothelial cells anchored by a basement membrane. The endothelial cells sense the hemodynamic and biochemical environment of the lumen and relay chemical signals to smooth muscle cells and fibroblast cells, allowing the aortic wall to adapt to both acute and chronic luminal changes.

The media is found superficially to the intima and is the most important contributor to biomechanical properties of the thoracic aortic wall. Specifically, the elastic fibers of the media, which are arranged in concentric layers and composed of extensively cross-linked tropoelastin, allow elastic fibers to be stretched and to recoil with minimal energy loss.^14^ Cross-linked elastin is formed primarily in the fetal and neonatal period and is insoluble with a half-life of 70 years, making it highly durable.^15^ Newly formed elastin in adults is of low quality, rendering any damage to elastic fibers largely irreversible. There is also a demonstrable relationship between the number of intact elastin layers and the pressure required to propagate dissection or cause aortic rupture.^16^ Elastic fibers are anchored to vascular smooth muscle cells via focal adhesions, as well as being supported by a scaffold of extracellular molecules, including fibrillin, integrins, and collagen. Vascular smooth muscle cells and fibroblasts also maintain the extracellular matrix environment by producing tropoelastin, collagens, and microfibrils, along with proteins that promote medial healing and remodeling, for example, matrix metalloproteinases (MMPs) and tissue inhibitors of metalloproteinases (TIMPs).

The outer section, the adventitia, is in communication with the nervous system and vasa vasorum, an external blood supply that also provides interaction with the immune system. The adventitia is composed primarily of fibroblasts that produce the fibrillar protein collagen. Unlike elastin, collagen has limited stretch or elasticity. However, it has a high resistance to biomechanical failure under peak stresses, meaning it plays an important role in protecting the aortic wall against rupture.^17^ Although aortic dissection represents a tear of the medial elastic fibers and preservation of the adventitial collagen, rupture denotes biomechanical failure of both adventitial collagen and medial elastic fibers.

## Categorizing Inherited Thoracic Aortopathies

Inherited aortopathies are generally categorized into 2 broad groups based on the clinical suspicion at presentation: syndromic or nonsyndromic aortopathy. Syndromic heritable thoracic aortic disease (sHTAD) denotes a varied group of genetically mediated conditions that are associated with systemic features of disease, aortic dilatation, and acute aortic events. The major sHTADs, as published by the GenTAC Registry (National *Registry* of Genetically Triggered Thoracic Aortic Aneurysms) results, include Marfan syndrome, Turner syndrome, Loeys-Dietz syndrome, and vascular Ehlers-Danlos syndrome.^18^ A number of other genetically mediated syndromes such as Shprintzen-Goldberg syndrome (*SKI* mutations) are associated with aortic dilatation, although in these conditions, progression to acute aortic events is exceptional or has not been reported (Table 2).

**Table 2.**
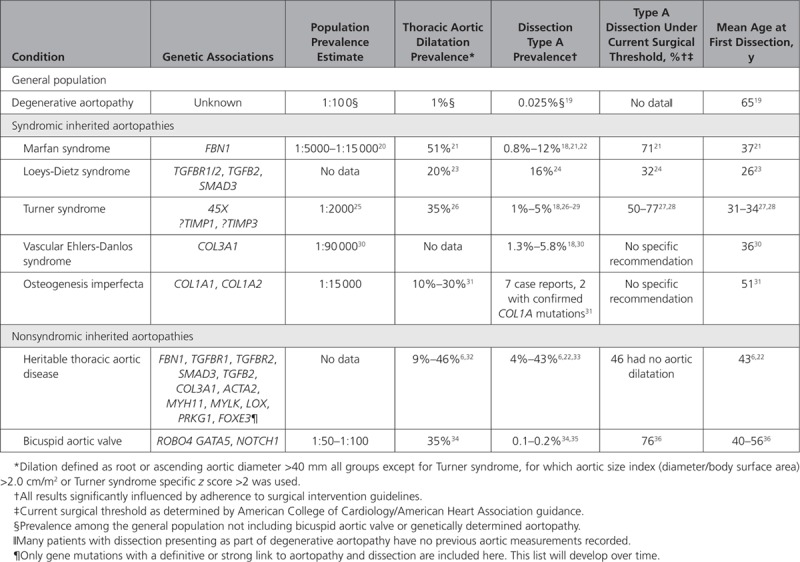
Epidemiological Data of the Major Inherited Thoracic Aortopathies Associated With Acute Aortic Events From Observational Studies Including Patients in the Post-2010 American College of Cardiology/American Heart Association Guideline Era

Non-sHTAD (nsHTAD) describes a familial form of aortopathy and dissections at a young age but without systemic features. In the past decade, studies exploring the underlying genetic mechanisms of the nsHTAD groups have allowed a superior understanding of how gene mutations influence aortopathy risk.^37^ A recent international consortium of experts in inherited thoracic aortic disease performed a validated semiquantitative analysis of the clinical and experimental data supporting a large number of genes associated with nonsyndromic thoracic aortopathy, concluding that there was sufficient evidence that *ACTA2*, *MYH11*, *MYLK*, *LOX*, *PRKG1*,and *FOXE3* confer a strong or definitive association with nsHTAD.^38^ Similarly, patients who have confirmed *TGFBR1*, *TGFBR2*, *SMAD3*, *TGFB2*, *COL3A1*, or *FBN1* mutations but do not meet phenotypic diagnostic criteria for a specific syndrome are classified as having nsHTAD. This list of genes will continue to evolve as new research elucidates and corroborates causative gene mutations. To deliver optimal management strategies for these patients, understanding the mechanisms by which gene mutations mediate disease and how these changes affect the risk of acute aortic events is crucial.

## Marfan Syndrome and Fibrillin

**Figure 2.**
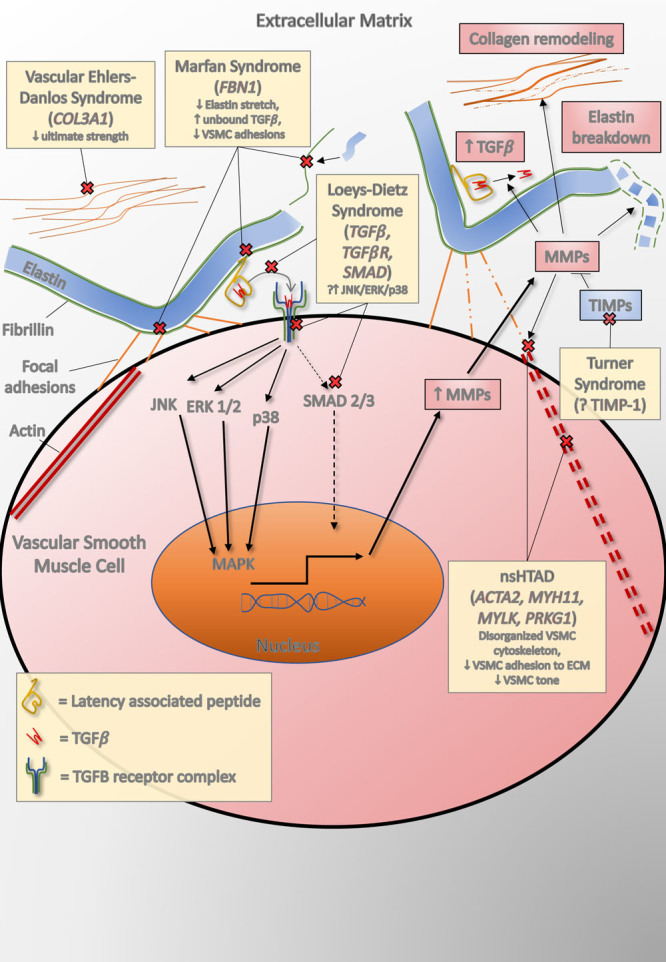
**Summary of the processes thought to be involved in thoracic aortopathy development.** The pathway affected as part of abnormal underlying processes in individual inherited thoracic aortopathies is highlighted. ECM indicates extracellular matrix; MMP, matrix metalloproteinase; nsHTAD, nonsyndromic heritable thoracic aneurysm and dissection; TGFβ, transforming growth factor-β; TIMP, tissue inhibitor of metalloproteinase; and VSMC, vascular smooth muscle cell.

Marfan syndrome is a clinical diagnosis conferred to patients who meet the revised international criteria, with the majority having mutations in *FBN1*,which codes for a glycoprotein called fibrillin-1 (Figure 2).^39^ Without intervention, patients with Marfan syndrome have an average life expectancy of ≈40 years; 80% of mortality is related directly to aortic dilatation, dissection, and rupture. In the modern era of regular aortic monitoring and prophylactic surgery, life expectancy has been much improved with reduced rates of acute aortic events (Table 2).^40^ Fibrillin-1 is the primary subunit of microfibrils, which are an important component of the aortic wall extracellular matrix. Fibrillin-1 plays a major role in the assembly and support of elastin by promoting adhesion to vascular smooth muscle cells through interaction with lysyl oxidase (encoded by *LOX*) and fibronectin, as well as enhancing elasticity through its microfibrillar structure (Figure 2).^41^ Changes in fibrilin-1 lead to reduced elastic fiber genesis in early life and to reduced elastic fiber adhesion to vascular smooth muscle cells, both of which compromise the biomechanical integrity of the aortic wall. Fibrillin-1 also has a role in maintaining aortic wall homeostasis by binding to latent transforming growth hormone-β–binding proteins, which sequester transforming growth factor-β (TGFβ; Figure 2), a molecule implicated in the pathogenesis of a number of inherited thoracic aortopathies.

## Loeys-Dietz Syndrome and TGFB Signaling

Patients with Loeys-Dietz syndrome carry mutations that affect the signaling of TGFβ (*TGFBR1*, *TGFBR2*, *TGFB2*, *TGFB3*, *SMAD2*, and *SMAD3*), resulting in aortic aneurysm, arterial tortuosity, hypertelorism, and bifid uvula with a propensity toward arterial dissection or rupture at a young age.^42^ The systemic phenotype in Loeys-Dietz syndrome varies widely, with patients with more severe systemic phenotypes, particularly vertebral artery tortuosity, being more likely to experience aortic events.^24^ Initial descriptions of patients with *TGFβR1* or *TGFβR2* mutations suggested a particularly low life expectancy of 26 years without intervention. However, a contemporary large international study demonstrates that the majority of patients are surviving past 70 years, albeit with an enduring high dissection prevalence (Table 2).^24^ TGFβ is part of the cytokine superfamily and is enmeshed in many biological processes, including aortic wall repair and homeostasis.^43^ TGFβ signaling follows 2 distinct and well-investigated pathways: small mothers against decapentaplegic (SMAD; canonical), or p38/extracellular signal-regulated kinase/c-Jun N-terminal kinase (noncanonical), the latter of which is thought to be detrimental to thoracic aortic aneurysm formation, although the precise mechanisms are still controversial (Figure 2).^44,45^

## Vascular Ehlers-Danlos, Osteogenesis Imperfecta, and Collagen

Pathological mutations in genes encoding type 3 (*COL3A1*, vascular Ehlers-Danlos) and, to a lesser extent, type 1 collagen (*COL1A1 and COL1A2*, osteogenesis imperfecta) are associated with syndromic forms of aortopathy, and patients with such mutations are at increased risk of acute aortic events (Table 2).^31^ Patients with vascular Ehlers-Danlos have a reduced life expectancy with an average survival of 51 years, with the majority of mortality arising from vascular complications. The arterial wall in vascular Ehlers-Danlos is particularly fragile with up to 6% experiencing dissection or rupture at an average age of 36 years, whereas individuals with osteogenesis imperfecta experience aortic dilatation relatively frequently (10%–30%), but reports of dissection are very rare (Table 2).^18,30,31^ Collagen is a fibrillar protein composed of a triple helix of α subunits that vary, depending on the type of collagen produced. The majority of collagen in the thoracic aorta is type 3 or 1, both of which confer robust ultimate strength, preventing rupture (Figure 2). Although the mechanism by which types 3 and 1 collagen mutations cause aortic wall vulnerability is intuitive, the role of collagen remodeling in other inherited thoracic aortopathies is unclear, with contrasting reports of increased and decreased concentrations in response to aneurysm formation.

## Turner Syndrome and TIMPS

Turner syndrome is the complete or partial loss of the second X chromosome, affecting ≈1 in 2000 women,^25^ although mosaicism can also occur and can affect men. It is associated with short stature, abnormal facies, and hypogonadism, with congenital heart disease affecting 25% to 50%, typically bicuspid aortic valve or coarctation of the aorta. There is a growing recognition that patients with Turner syndrome are at risk of thoracic aortopathy,^27^ with >35% reported as dilated and up to 5% experiencing dissection or rupture (Table 2).^18^ Similar to other inherited thoracic aortopathies, the precise mechanism of disease in Turner syndrome remains elusive, although recent genetic studies have implicated TIMP1 and TIMP3.^46^ Haploinsufficiency of TIMP1 increased the risk of aortopathy 10-fold, and concomitant TIMP3 mutation augmented that risk to 18-fold.^26^ Given the evolving role that TIMPs have in aortic remodeling and aneurysm formation, TIMP1 and TIMP3 may be important targets for future therapeutic and biomarker translation in inherited aortopathies generally.

## Nonsyndromic Heritable Thoracic Aortic Disease

nsHTAD represents a relatively heterogeneous cohort of patients with a family history of aortic dissection at a young age with no clear syndromic features suggestive of a connective tissue disease. Nonsyndromic aortopathy is increasingly recognized as an important clinical entity, seen in up to 89% of aortopathy cohorts.^47^ The underlying genetic causes of nsHTAD include mutations in genes encoding members of TGFβ signaling pathway (*TGFBR1*, *TGFBR2*, *TGFB2*, *SMAD3*); the structure and integrity of the aortic wall extracellular matrix, including *FBN1* (described earlier); *COL3A1* and lysyl oxidase (*LOX*); or those affecting the function of smooth muscle cells, including actin (*ACTA2*), myosin (*MYH11*), myosin light chain kinase (*MYLK*), and protein kinase cGMP-dependent 1 (*PRKG1*). Taken together, causative mutations can be found in only ≈20% of nsHTAD cases, leaving many without a genetic diagnosis.^32^ In probands with a confirmed mutation, many have mutations in *FBN1* without meeting revised Ghent criteria to diagnose Marfan syndrome.^47^ Furthermore, up to 35% have mutations in *SMAD3* and 14% have *ACTA2* mutations, whereas pathological mutations in *MYH11*, *MYLK*, *PRKG1*, *LOX*,and other less well-depicted genes are limited to a smaller number of families.^33,48,49^ In a cohort of patients experiencing acute aortic events with confirmed pathological mutations, the average age at the time of the event was 41 years, with only 54% having aortic root aneurysms (Table 2).^32^ Those with pathological mutations are more likely to experience aortic intervention or dissection at a young age, particularly those <50 years of age, whereas those >60 years of age are much less likely to carry pathological mutations.^33,47^ In patients with nsHTAD with a pathogenic mutation, many mutations cause disease by affecting the function of vascular smooth muscle cells.^33,47^ Vascular smooth muscle cells are the primary cell of the medial layer, providing contractile stress (tone) and anchoring the elastic fibers of the extracellular matrix, which confers overall stability. The intracellular structure of vascular smooth muscle cells and tone generation are governed by smooth muscle cell–specific α-actin and myosin, the function of which can be disrupted in the vascular smooth muscle cell mutations, leading to increased cell death, decreased aortic wall tone, and reduced extracellular matrix stability (Figure 2).

## Bicuspid Aortic Valve Aortopathy, Abnormal Aortic Development, and Abnormal Flow

Bicuspid aortic valve affects 0.5% to 2% of the population and denotes an aortic valve composed of 2, rather than 3, aortic leaflets (Figure 1).^50^ Patients with bicuspid aortic valve are at increased risk of aortic stenosis and regurgitation and are significantly more likely to experience ascending aortic dilatation and acute aortic events (Table 2). The mechanisms that predispose patients with bicuspid aortic valve to aortopathy are still emerging, with evidence supporting both biomechanical and genetic causes. Multiple studies have demonstrated the important influence that abnormal thoracic aortic flow patterns have on underlying aortic wall TGFββ pathway signaling, elastin breakdown, dilatation location, and the risk of aortic events.^51,52^

There are sex and racial differences in the prevalence of bicuspid aortic valve, with men and white individuals the most likely to be affected.^53^ Furthermore, there is clustering of affected individuals within families. Taken together, these strongly suggest the presence of an inherited disease process. Yet, similar to nsHTAD, in the majority of cases, no definite genetic cause can be found.^54^ In those patients with mutations known to affect normal aortic development, for example, *FBN1* or TGFβ βpathway mutations, there is an increased prevalence of bicuspid aortic valve, suggesting an inherent link between bicuspid aortic valve formation and thoracic aortopathy in these groups.^55^ Furthermore, there is a growing number of reports describing specific mutations in patients with bicuspid aortic valve and aortopathy but without the systemic features of connective tissue disease, for example, *ROBO4* and *NOTCH1*, which affect endothelial permeability and endocardial/endothelial differentiation, respectively, although the link between *NOTCH1* and aortic events is not well described.^56,57^ Overall, although bicuspid aortic valve is an independent risk factor for developing a progressive aortopathy, it is clear that the risks are compounded by a spectrum of genetic variants that affect how the thoracic aortic wall develops and remodels. In clinical practice, the ambiguity surrounding the genetic influence on acute aortic event risk can make deciding when to offer familial aortic screening and genetic evaluation challenging.

## Clinical Evaluation and Genetic Testing in Suspected Inherited Aortopathy

When patients and their families are being investigated for suspected inherited thoracic aortopathy, important consideration will be given to whether genetic testing is indicated and how to counsel patients about the potential implications. Here again, it is useful to split into syndromic and nonsyndromic categories. In a patient presenting with suspected sHTAD, imaging and genetic investigations vary significantly according to the proband phenotype (summarized in Table 3). Familial testing in relatives will be guided by a genetics specialist and will be focused on those family members with phenotypic features suggestive of the condition.

**Table 3.**
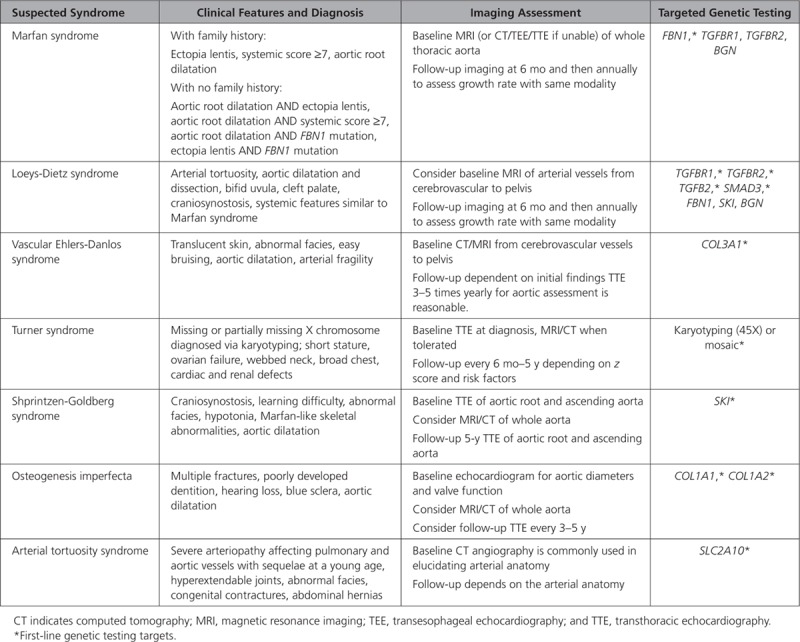
Summary of Diagnostic Criteria, Imaging Assessment, and Targeted Genetic Testing for Syndromic Inherited Aortopathies

**Figure 3.**
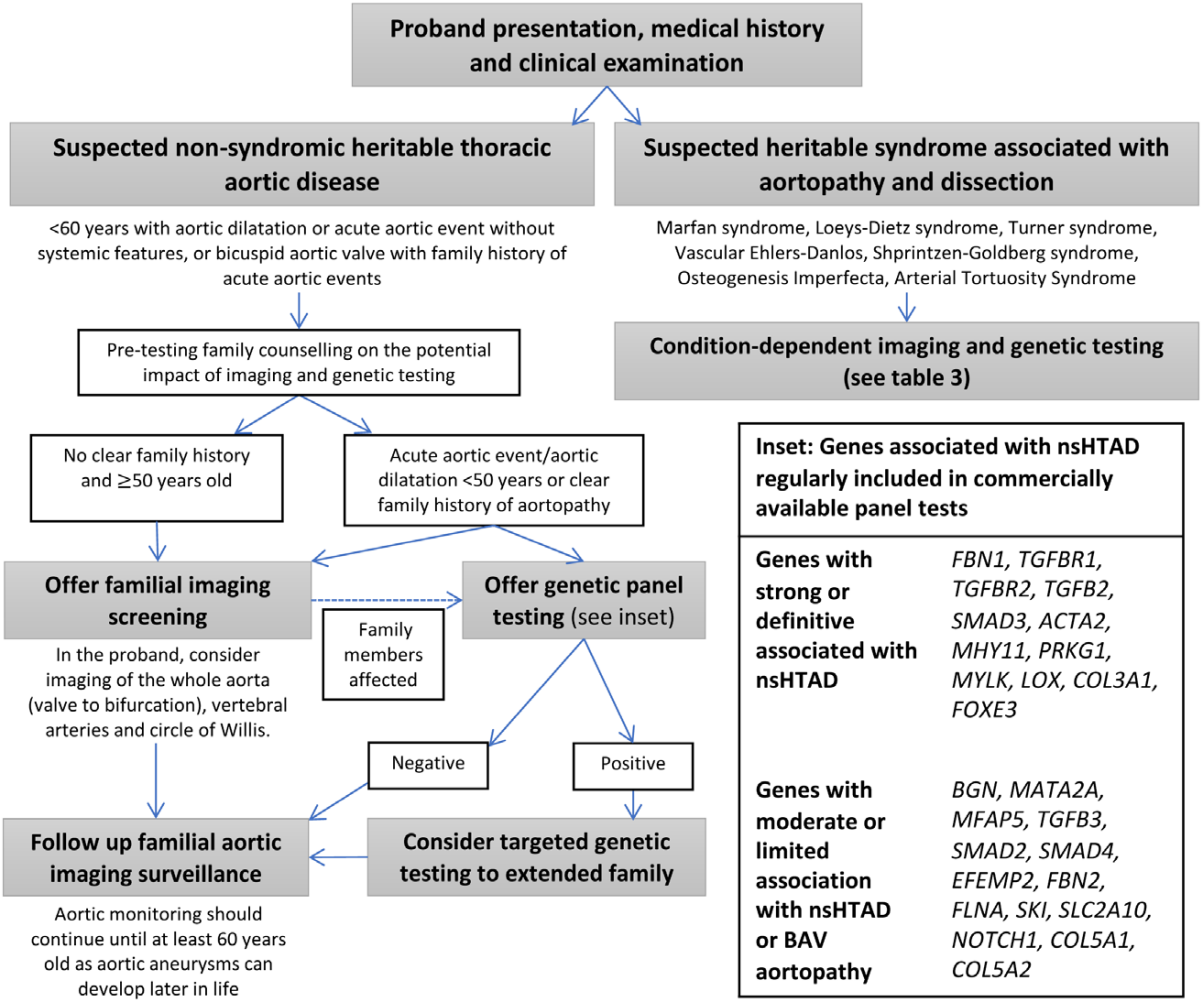
**Summary of the management for imaging, family screening, and counseling management for inherited thoracic aortopathies.** First-line genetic testing targets are in bold. BAV indicates bicuspid aortic valve; and nsHTAD, nonsyndromic heritable thoracic aortic disease.

Managing patients presenting as the proband for nsHTAD can be challenging, with a degree of uncertainty often surrounding diagnosis, prognosis, and how to investigate family members. First, careful elucidation of any family history of acute aortic events, including sudden unexplained death at a young age, is important in establishing a clear heritable aortopathy. Echocardiographic screening of the aorta in first-degree relatives can be a useful and affordable tool for establishing the prevalence of aortic dilatation within the family, both solidifying a diagnosis of an inherited aortopathy and identifying at-risk family members who will require more regular aortic surveillance (Figure 3). Second, because nsHTAD with aortic dilatation or dissection in patients <50 years of age has a stronger likelihood of identifiable genetic mutations, offering genetic testing in these patients is reasonable even in the absence of a clear family history (Figure 3).^32,47^ When a heritable thoracic aortopathy is suspected, genetic testing should be considered carefully and discussed with the family, usually by a geneticist specialist.

Genetic screening in nsHTAD has developed over the past decade and can be beneficial when applied in the right context by providing more personalized selection for aortic surveillance and the timing of prophylactic aortic surgery and conveying a better understanding of the risks of pregnancy and to future offspring. In the clinical setting, this genetic testing is best performed when positive results are actionable by adding meaningful prognostic information affecting the patient’s medical care and influencing important clinical decisions, for example, the timing of prophylactic aortic replacement surgery. Families should be aware that a clear genetic cause can be found in only ≈20% of cases, which may lead to a degree of uncertainty.^33^ In these cases, a robust mechanism for monitoring family members, whether apparently affected or not, will be required because aortopathy may not develop until later in life (Figure 3).^58^ The majority of commercially available gene panels across the United States and Europe also include targeted sequencing of genes with an association with thoracic aortopathy that is less well established (Figure 3). Although there is a role for expanded genetic testing, particularly in the context of gene discovery research, prospective counseling on the consequences of uncertain results and follow-up aortic monitoring will be crucial when supporting families in this scenario. Improved understanding of the gene-disease aortic risk association and novel gene discovery are going to be vital to improving the care received by these families with inherited aortopathies. To move toward the eventual goal of personalized gene-adapted management, longitudinal international collaboration with the pooling of affected patients will be essential to overcoming the lack of power consequent to the rarity of gene-positive disease. Until then, treatment, management, and counseling of patients with a confirmed inherited aortic disease are based on reducing the risk of acute aortic events through an understanding of biomechanical and biological principles.

## Biomechanical Principles of Aortopathy Risk

Acute aortic events represent the biomechanical failure of the layers of the aortic wall and occur when the wall stress exceeds the ultimate rupture strength. Increased aortic wall circumferential stress is understood to be related to an increase in blood pressure and aortic diameter or a decrease in aortic wall thickness through the Law of Laplace. Expansion of aortic diameter with sustained blood pressure, as with aortic dilatation, increases wall stress and subsequently the risk of acute aortic events. This risk is reflected in the substantial increase in aortic event rate seen in ascending aortic aneurysms >60 mm. Similarly, increased blood pressure caused by raised systemic vascular resistance—for example, coarctation, obesity, or essential hypertension—raises wall stress and consequently the risk of acute aortic events.^35^ Likewise, pregnancy is associated with increased cardiac output and hormonal changes that, in the context of aortopathy, can lead to an irreversible increase in aortic root diameter and a higher likelihood of acute aortic events.^59^ This risk is reflected in current international counseling guidance that categorizes all inherited thoracic aortic diseases as at least modified World Health Organization risk group II (small increase risk of maternal mortality or moderate increased risk of morbidity) with a reasonable proportion in the highest risk category IV (extremely high risk of maternal mortality or severe morbidity), depending on the condition and degree of prepregnancy aortic dilatation (Table 4).^60^ Prepregnancy counseling is therefore crucial for women of child-bearing age with aortopathy.

**Table 4.**
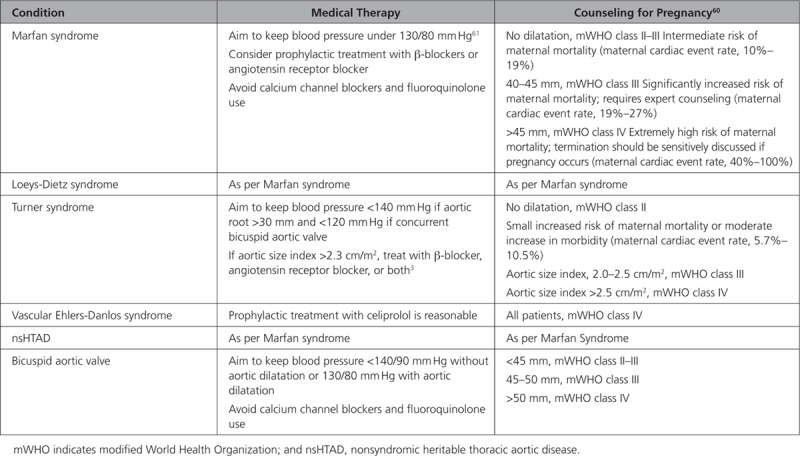
Summary of Current Treatment Guidance for Inherited Thoracic Aortic Diseases

Under normal circumstances, when blood pressure is sustainably raised, the aortic wall remodels and increases in thickness, thereby keeping circumferential stress relatively constant. However, there is emerging evidence that remodeling that results in reduced compliance of the aortic wall is a worrying feature of aortopathy.

## Aortic Stiffness

Aortic stiffness is a marker of how easily the aortic wall distends with applied pressure. With noninvasive two-dimensional aortic imaging (computed tomography, magnetic resonance imaging [MRI], or ultrasound), aortic compliance can be assessed by the use of strain, the increase in the aortic wall length as a proportion of the baseline length. Strain can be measured in the circumferential and longitudinal directions, with distensibility representing circumferential strain divided by pulse pressure.^62^ Alternatively, aortic stiffness can be inferred from how quickly the pulse wave propagates through the aorta, known as pulse-wave velocity, which is measured with applanation tomography or extrapolated from four-dimensional flow sequences on MRI.

Aortopathy in Marfan syndrome, Loeys-Dietz syndrome, Turner syndrome, and nsHTAD is associated with increased aortic stiffness.^63,64^ Ex vivo biomechanical testing of dilated aortas demonstrates that increased stiffness appears to be attributable to higher collagen and lower elastin content, making aortic stiffness a proxy marker of wall remodeling, which may be apparent well before aortic diameters begin to increase.^14^ Consistently, the aortic stiffness increases with age, and a steep rise in stiffness is seen after 55 years of age, coinciding with the natural decreases in elastin. There is also a strong relationship between increasing diameter and increasing aortic stiffness. In Marfan syndrome, aortic stiffness is increased in those with either dilated or nondilated aortas. More important, reduced longitudinal aortic strain is associated with increased aortic event rates in Marfan syndrome after adjustment for aortic diameter.^62^ Larger studies are required to corroborate the role of aortic stiffness in identifying high-risk aortopathy after adjustment for dilatation in connective tissue disorders and bicuspid aortic valve.

In patients whose aorta is inherently weak or remodels abnormally, as with inherited aortopathies, increases in blood pressure, systemic vascular resistance, and aortic stiffness are detrimental and should be addressed. In the past decade, significant research effort has focused on improving our understanding of how medical therapies can affect outcomes through their influence on biomechanical and biological properties.

## Medical Treatment of Thoracic Aortopathies

Conventional therapies have focused on reducing aortic wall stress by lowering blood pressure and systemic vascular resistance. The 2017 American College of Cardiology/American Heart Association guideline for the treatment of hypertension recommends β-blockers as first-line treatment in all thoracic aneurysm disease.^65^ The European Society of Cardiology/European Society of Hypertension 2018 guideline on the management of hypertension in aortic diseases recommends strict blood pressure control to <130/80 mm Hg.^61^ The evidence for a benefit for presumptive β-blocker therapy stems from a reduced rate of aortic dilatation associated with a reduction in blood pressure and acute aortic events in nonhypertensive patients with Marfan syndrome treated with propranolol compared with control subjects.^66^ Angiotensin receptor blockers, which target the AT1 receptor with a consequent reduction in TGFβ signaling, have also been investigated as a potential therapeutic target, given the promising results in animal models. Large randomized controlled trials conducted so far have not found an effect of angiotensin receptor blockers on clinical outcome compared with the protection provided by β-blocker therapy.^67–69^ There is, however, some recent promise for the ability of irbesartan to reduce aortic growth rate when added to current treatment.^70^ Although currently there is not enough evidence to recommend angiotensin receptor blocker therapy over β-blocker therapy, its use is reasonable when β-blocker therapy is poorly tolerated. Further elucidation of the mechanisms governing TGFβ activity in Marfan syndrome is required.

Although there is some evidence base for β-blocker or angiotensin receptor blocker therapy in Marfan syndrome, calcium channel blockers appear to be detrimental. Both murine models and a case-controlled analysis of humans with Marfan syndrome demonstrated an increased risk of dissection/rupture for treatment with channel blockers.^71^ Similarly, fluoroquinolone use appears to be linked to aortic aneurysm formation and dissection in the general population, possibly through an imbalance of TIMPs and MMPs (Figure 2) and reduced collagen.^72,73^ Although medical therapy in Marfan syndrome is relatively well studied, the evidence for presumptive blood pressure–lowering therapy in other inherited aortopathies is scarce.

In vascular Ehlers-Danlos syndrome, treatment with celiprolol (a selective B_1_ blocker) was associated with a reduced event rate, although there was a small increase in brachial blood pressure, suggesting that blood pressure was not the mediator of aortic risk in these patients.^74^ The guidance on the medical management for other inherited thoracic aortopathy is extrapolated from studies on Marfan syndrome. Given the diverse mechanisms of aortic wall vulnerability in inherited aortopathies (Figure 2), studies that examine the role of both established and novel medical therapies in specific inherited aortopathies, for example, the role of angiotensin receptor blocker therapy in Loeys-Dietz syndrome, are required to provide improved and targeted therapy.

Given that between 30% and 80% of acute aortic events in those with inherited aortopathy occur under surgical thresholds (Table 2), novel methods of identifying patients with thoracic aortic disease at high risk of dissection or rupture are urgently required.

## Blood and Tissue Markers of Inherited Thoracic Aortopathy

Although understanding of the processes that govern the different inherited thoracic aortopathies has progressed, translating these findings into therapeutic targets or robust markers of thoracic aortic disease severity is still in the early stages. Blood markers are easy to obtain and relatively cheap, making them an attractive option if a reliable, sensitive, and specific test can be found. Research efforts to uncover such a suitable blood marker have focused primarily on mediators and markers of aortic wall destruction. Here, the pathways targeted as part of translational work performed thus far are summarized, but a detailed overview of each family of molecules is beyond the scope of this review.

### Plasma TGFβ

As outlined, murine models of both Marfan syndrome and Loeys-Dietz syndrome have implicated TGFβ signaling, particularly the noncanonical pathway, as being detrimental (Figure 2). Blood measurement of TGFβ is an attractive biomarker both for linking to disease progression and as a research tool in measuring effects of TGFβ-modifying treatments (eg, TGFβ response to angiotensin receptor blocker therapy). Progress toward translation has been hampered by difficulties in reliably measuring active TGFβ1 in the blood. Platelets are TGFβ1 rich, and degranulation, as occurs in serum preparation and when exposed to high shear stress as part of sampling, can contribute to 90% of TGFβ1 levels and therefore mask any changes caused by pathological processes.^75^ Optimal blood-taking methodology that reduces the amount of platelet degranulation has been well described,^76^ with many studies not reporting venesection techniques or measuring concomitant platelet activation. When concomitant platelet activity is assessed, no difference can be found in plasma TGFβ1 concentrations between patients with Marfan syndrome with dilated aortic roots or previous surgery and control subjects.^77^ Given the evidence for its role in the progression of aortopathy, active TGFβ1 could be a useful marker of disease progression and a guide to effective medical treatment. However, more research is required to ensure reliability (adjusting for platelet activation) and international consistency. Furthermore, because TGFβ1 is abundant throughout the body, whether plasma concentrations specifically reflect aortic wall activity and predict disease progression remains to be validated.

### Matrix Metalloproteinases

MMPs are a family of 23 separate molecules that share the characteristic feature of a zinc-binding catalytic area and are secreted by vascular smooth muscle cells, fibroblasts, and immune cells in response to chemical or biomechanical stress. The primary role of these MMPs is to cleave and digest extracellular proteins such as elastin, collagen, and integrins as part of aortic wall repair, remodeling, and homeostasis. Under pathological conditions, the unchecked activity of specific MMPs can be detrimental to the cardiovascular system by causing excess destruction of vascular wall components. Murine studies have specifically implicated MMP-2 and MMP-9 in thoracic aortic aneurysm formation, and they are the most promising targets for translation as markers and potential modulators of disease.^78,79^

Efforts have been made to use MMP-2 and MMP-9 as prognostic biomarkers for thoracic aortic disease severity. Human induced pluripotent stem cell line study of patients with Marfan syndrome demonstrates increased MMP-9 levels compared with reference cell lines.^80^ As with TGFβ1, translation of MMPs into reliable biomarkers of aortic wall remodeling is attractive. However, blood MMP-9 and MMP-2 concentrations are sensitive to caffeine and the collection method (citrate deemed most stable) and require quick processing (within 2 hours) to prevent artifactual changes in quantification.^81,82^ Appropriately measured plasma MMP-2 concentrations have been linked to increased aortic diameter and repeatably linked to increased aortic stiffness, although no studies to date have made links to outcomes.^83,84^ Given their association with abnormal aortic function and the mechanism of action, MMPs have been a target for translation of medical therapy. Doxycycline, a generalized inhibitor of MMPs, can prevent aneurysm formation and preserve elastic fiber integrity and aortic biomechanics in animal models.^85^ However, when translated to human abdominal aortic aneurysms, 100 mg doxycycline over 18 months resulted in a small but significant increase, rather than decrease, in aortic growth rate compared with placebo.^86^ Although MMPs appear to play a role in aortic wall pathology, their precise role remains to be determined. Progress may be made by improving our understanding of how MMPs are moderated through their naturally occurring inhibitors.

### Tissue Inhibitors of MMP

TIMPs are a family of 4 naturally occurring and nonspecific MMP inhibitors found in a wide variety of biological systems, including the vasculature. They function by interacting with the active site of target MMPs and deactivating pro–MMP-2 and -9, working in concert to promote vascular homeostasis without excess destruction. Under pathological circumstances, MMP activity unchecked by TIMP is thought to promote abnormal remodeling.

Turner syndrome is an interesting inherited thoracic aortopathy with a high incidence of bicuspid aortic valve that can be used to study TIMP1 as a mediator of thoracic aortopathy. The gene is located on the X chromosome, 1 copy of which is lost in many cases of Turner syndrome.^26^ Moreover, because bicuspid aortic valve disease is male predominant, some have speculated that having 2 sets of X chromosomes is in some way protective against bicuspid aortic valve and aortopathy. Although TIMP1 may be implicated in inherited aortopathic disease, there is little evidence that plasma TIMP1 concentrations reflect gene copy number or mirror aortic pathological processes.^46,87^ Some researchers have instead focused on the ratio of MMPs to TIMPs, giving a picture of an overall balance between remodeling and destruction and finding increased ratios in those with bicuspid aortic valve, although these findings require further validation.^88^

Overall, the roles of TIMP1 as a mediator of remodeling make an attractive target for biomarker translation, particularly when coupled with MMP-2 and MMP-9 to give a representation of remodeling activity balance. However, as with many biomarkers, both MMPs and TIMPs are involved in many biological processes; therefore, blood concentrations may not be specific enough to represent thoracic aortic remodeling.

### Desmosine

A promising development in biomarker research for thoracic aortopathies lies in plasma desmosine, a rare molecule that is necessary for cross-linking tropoelastin. Desmosine appears to increase with age and is elevated in patients with chronic obstructive pulmonary disease, reflecting elastin breakdown.^89^ Desmosine has very few other functions in humans, potentially making it a specific marker of aortic wall elastin breakdown.^90^ In abdominal aortic aneurysms, increasing plasma desmosine concentrations were associated with aortic diameter and an increasing likelihood of aortic events, after adjustment for diameter.^91^ Although the breakdown of elastin is not specific to a single inherited aortopathy, it represents an end point thought to correlate with biomechanical weakness and a tendency toward failure. Studies exploring the utility of plasma desmosine concentrations to predict dissection or rupture in thoracic aortopathy would be highly valuable.

Although a significant amount of research has focused on novel blood biomarkers, the evidence supporting their role as a complementary marker of aortopathy risk is relatively weak, with most targets being involved in many processes throughout the body. Therefore, blood concentrations are unlikely to reflect changes at the aortic wall level. In this regard, molecular imaging techniques, which allow the direct visualization of biological processes at specific anatomic locations, may prove beneficial.

## Molecular Markers of Aortopathy

Advanced imaging techniques with specific tracers have the potential to localize and quantify (patho)biological processes occurring within the body. Molecular imaging with a radioactive molecule or a targeted smart contrast agent can be used to provide combined functional and structural imaging with a range of imaging techniques such as positron emission tomography, MRI, or computed tomography. The anatomic visualization of molecular activity allows organ-specific assessment of these processes, improving accuracy and specificity.

### Elastin-Specific MRI Contrast Agent

**Figure 4.**
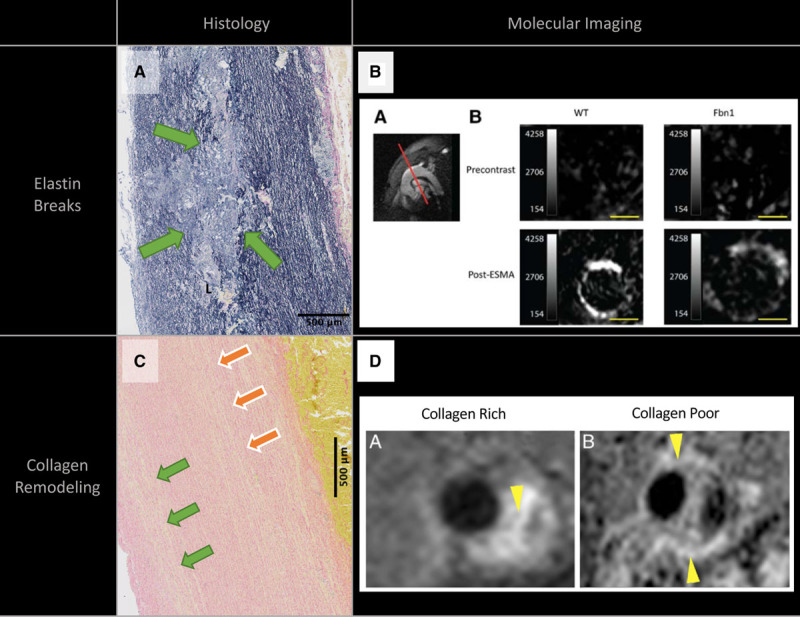
**Emerging molecular imaging methods for identifying high-risk aortopathy.** Histological sections from a patient with Marfan syndrome who underwent elective aortic root replacement for dilated aorta highlighting known pathological end points in thoracic aortic disease (**A** and **C**) and corresponding examples of molecular imaging for each disease process (**B** and **D**). **A**, Elastin van Gieson staining demonstrating elastic fiber breaks (green arrows). **B**, Uptake of elastin-specific contrast agent was performed in control (wild-type [WT]) and FBN1^C1039G/+^ mice at the level of the ascending aorta with reduced uptake in the ascending aorta of FBN1^c1039G/+^ mice, suggesting lower elastin content and more elastin breaks. Adapted from Okamura et al.^93^ Copyright © 2014, American Heart Association, Inc. **C**, Picrosirius red staining for type I and III collagen with areas of reduced uptake in the media and adventitia (green and orange arrows). Collagen-rich (**left**, yellow arrow) and collagen-poor (**right**, yellow arrows) abdominal aortic aneurysms in an angiotensin II infusion/transforming growth factor-β neutralization mouse model. ESMA indicates elastin-specific magnetic resonance imaging contrast agent. Reprinted from Klink et al^94^ with permission. Copyright © 2011, Elsevier.

Complementing the blood biomarker desmosine, an elastin-specific MRI contrast agent (ESMA) is now being investigated as a way of highlighting the aortic wall, with reduced uptake associated with decreased elastin content and aortic wall disease.^92^ In the murine model of Marfan syndrome, in vivo ESMA MRI quantification demonstrated reduced elastin compared with wild-type mice, a finding corroborated on histology (Figure 4).^93^ The ESMA MRI protocol has now been tested on anesthetized pigs, demonstrating good signal-to-noise ratio of the aortic wall and excellent colocalization of ESMA to elastin in electron microscopy. There are currently no studies using ESMA MRI in humans.

### Aortic Microcalcification and ^18^F-Sodium Fluoride

Vascular medial microcalcification is the pathological accumulation of hydroxyapatite crystals within the media of large arteries and has been strongly linked to adverse cardiovascular outcome. It occurs through multiple mechanisms that are separate from the intimal calcification that occurs in in atherosclerosis. Vascular medial microcalcification is understood to occur via 3 primary mechanisms: calcium deposition on damaged elastin, excess intracellular calcium released by stressed smooth muscle cells into vesicles or through cell death, or pathological osteogenic phenotype switching by vascular smooth muscle cells.^95^ Because the presence of medial microcalcification represents ongoing pathological processes, noninvasive detection and quantification could be a valuable marker of aortic wall disease. ^18^F-sodium fluoride is a molecular tracer that binds to hydroxyapatite, thus allowing noninvasive detection and quantification of aortic wall microcalcification.^96^ A recent study investigating the utility of ^18^F-sodium fluoride to predict clinical events in abdominal aortic aneurysms found that patients in the highest tertile of aneurysmal ^18^F-sodium fluoride uptake had the quickest growth rate and an increased incidence of surgical repair or rupture.^97^ Given the role that medial microcalcification plays in many common end points of pathological change of ascending aortopathy (elastin calcification, vascular smooth muscle apoptosis, extracellular matrix calcium deposition) and the promising results toward abdominal aortic aneurysms prognostication, it would appear to be an ideal candidate for translation in inherited thoracic aortopathy.

## Aortic Inflammation and ^18^F-Fluorodeoxyglucose

Unlike abdominal aortic aneurysms, the inflammatory process in thoracic aortopathy is less pronounced, with only weak associations between immune system activation and thoracic aneurysm formation. Indeed, there is some association with T cell–mediated medial degeneration, but this appears to be a late manifestation of the thoracic aneurysm formation process. ^18^F-fluorodeoxyglucose is a positron emission tomography radiotracer that nonspecifically highlights areas of high glucose metabolism, commonly found in areas with a high concentration of inflammatory cells or metabolically active cells. In abdominal aortic aneurysms, high ^18^F-fluorodeoxyglucose positron emission tomography imaging has been linked to increased cardiovascular events in general, as well as correlating with increased inflammation and MMPs in abdominal aortic aneurysms.^98^ However, at present there are no studies directly assessing the relationship between ^18^F-fluorodeoxyglucose uptake in the thoracic aorta and clinical outcomes focused on those with inherited aortopathy.

## Aortic Disease Activity and Novel Radiotracers

Although immune infiltration is not prevalent in inherited ascending aortopathy, vascular smooth muscle cells produce damaging MMPs under pathological conditions that contribute to medial degeneration and aortic remodeling. In the angiotensin II model of abdominal aortic aneurysm, the MMP-specific radiotracer ^99m^Tc-RP805 correlated with aneurysm size and predicted rupture or aneurysm formation.^99^ Although promising and relevant to ascending aortopathy, there are no reported applications in inherited thoracic aortopathy. Similar to MMP radiotracers, preclinical agents are being developed for visualizing collagen (Figure 4)^94^ and glycosaminoglycans,^100^ which may have potential translation into thoracic aortic pathology, but translating these agents into human study will require significant work.

## Conclusions

Inherited thoracic aortopathies encompass all patients born with an inherent risk of developing thoracic aortopathy. A combination of an abnormal hemodynamic profile and genetic abnormalities leading to aortic wall weakness predisposes these patients to life-threatening aortic dissection or rupture. Current management relies on aortic diameter, along with a few clinical risk factors, to decide on whether surgical intervention is required. However, there is international recognition that better models for predicting and preventing acute aortic events is required. Our understanding of the mechanisms underpinning inherited aortopathies has improved substantially, although major uncertainties still surround the identification and management of some inherited aortopathies, particularly nsHTAD. Continuing to develop large international collaborative research efforts is vital to improving management strategies for imaging, treatment, genetic screening, and counseling of these individuals. Novel therapies and biomarkers of thoracic aortopathies are urgently required. However, thus far, translating improved mechanistic understanding into reliable biomarkers or therapies has proved difficult.

TGFβ, MMPs, and TIMPs are all implicated in thoracic aortic wall disease. However, blood biomarker research has been hampered by variability in sampling and processing methodology and their nonspecific nature. The molecule desmosine, on the other hand, appears to be a specific marker of elastin breakdown and warrants further attention.

Aortic diameter remains the primary indicator for surgery despite known issues with its sensitivity and specificity for predicting aortic events. Alternative imaging markers such as aortic stiffness may provide complementary information on aortic risk. Molecular imaging is a promising technique combining molecular biology with anatomic colocalization. Visualizing aortic elastin and inflammation by ESMA and ^18^F-fluorodeoxyglucose positron emission tomography imaging, respectively, is an emerging technique for identifying high-risk inherited aortopathies that needs validation in human thoracic aortic aneurysms. Other radiotracers that target aortic wall collagen, microcalcification, MMPs, and glycosaminoglycans are at various stages of preclinical development and will be essential to improving our understanding of inherited aortopathies.

## Sources of Funding

This work was supported by the British Heart Foundation (FS/19/15/34155 to A.J. Fletcher, Dr Walker, and Professor Newby; FS/18/31/33676 to M.B.J. Syed; CH/09/002 and RE/18/5/34216 and RG/16/10/32375 to Dr Newby) and Wellcome Trust (WT103782AIA to Dr Newby).

## Disclosures

Dr Fletcher has received a 1-year salary as part of a Siemens imaging fellowship. The other authors report no conflicts.
